# Supplementary material for: Ultrafast evolution and transient phases of a prototype out-of-equilibrium Mott–Hubbard material
Source: Nat Commun. 2017 Jan 9;8:13917. doi: 10.1038/ncomms13917 (PMC5228036; doi:10.1038/ncomms13917)
Supplement: Supplementary Information — Supplementary Figures 1-10, Supplementary Notes 1-5 and Supplementary References [file ncomms13917-s1.pdf]

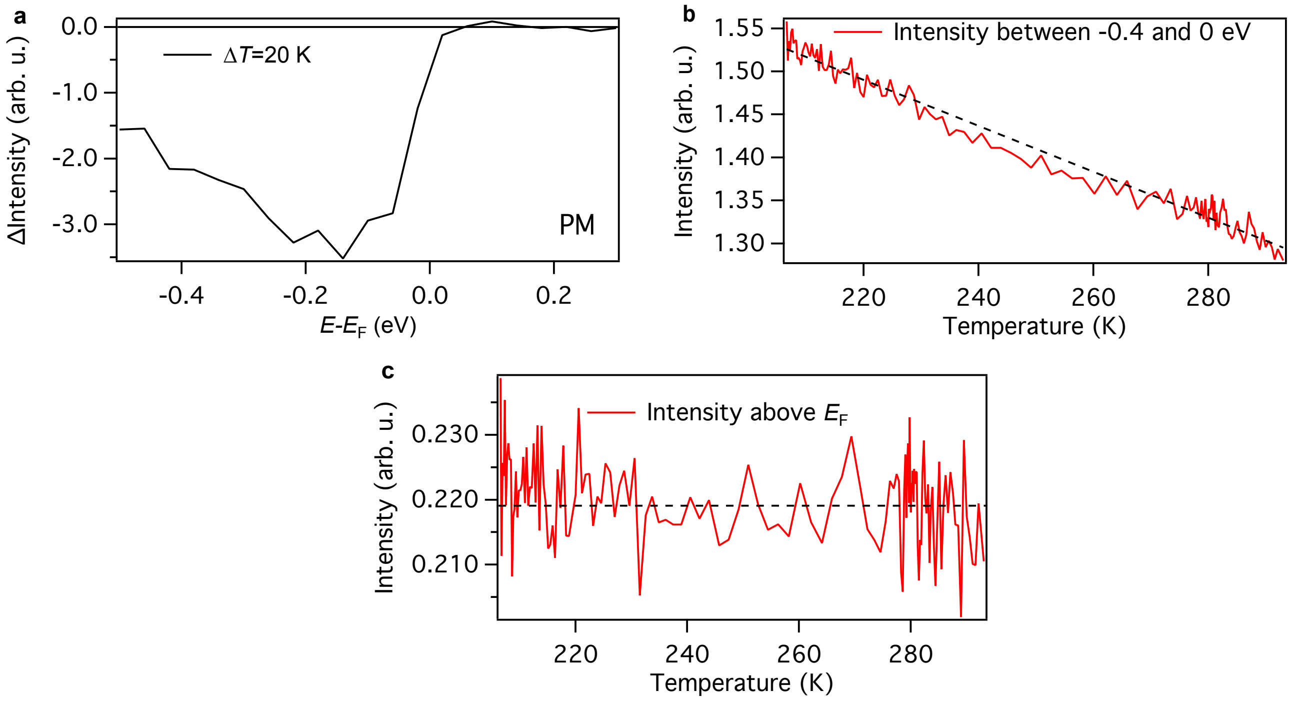

Supplementary Figure 1: Equilibrium photoemission spectra versus temperature for the PM phase a): Equilibrium photoemission spectra difference between 200 K and 220 K for V_2O_3 (PM phase). **b)**: Intensity between -0.4 and 0 eV versus temperature, which corresponds to the quasiparticle weight. It decreases linearly with temperature in the range observed. **c)**: Intensity above the Fermi level versus temperature, no clear trend is visible.

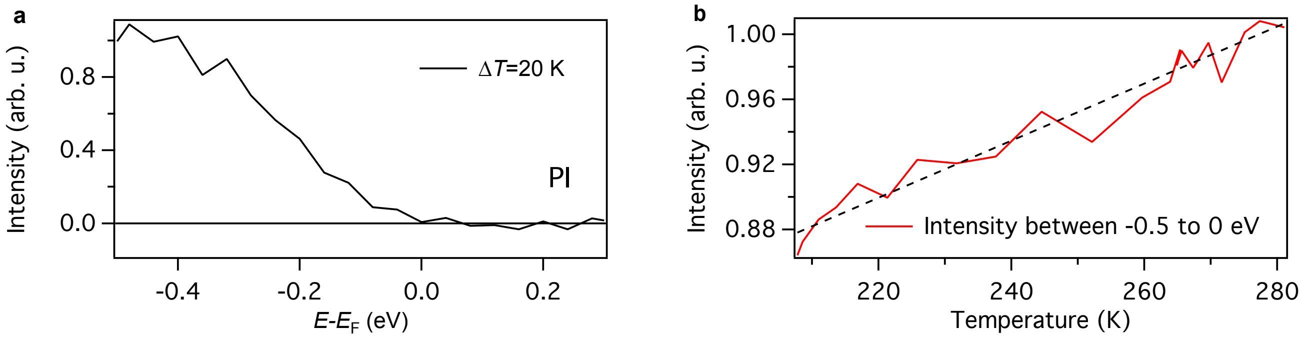

Supplementary Figure 2: Equilibrium photoemission spectra versus temperature for the PI phase a): Equilibrium photoemission spectra difference between 200 K and 220 K for Cr doped V_2O_3 (PI phase). **b)**: Intensity between -0.6 and 0 eV versus temperature. It increases linearly with temperature in the range under consideration.

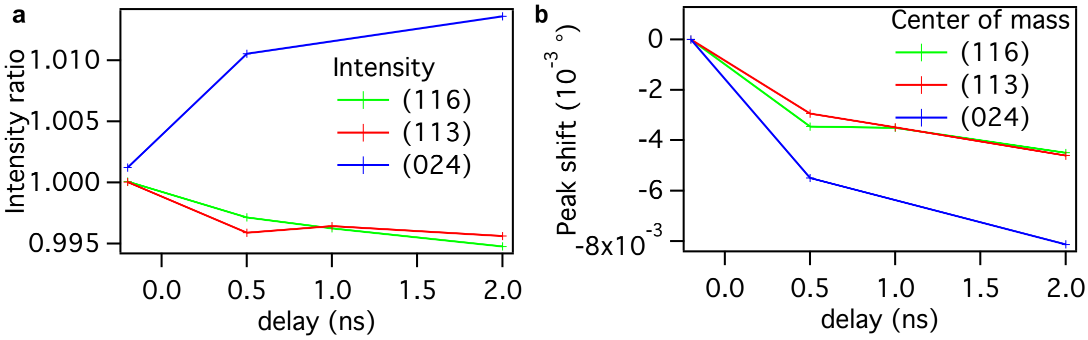

Supplementary Figure 3: Heating effects seen with Time-resolved X-ray diffraction Time-resolved X-ray diffraction measurement on pure V_2O_3 (PM phase) using 100 ps resolution from synchrotron radiation. The intensity and center of mass of the rocking curves are represented.

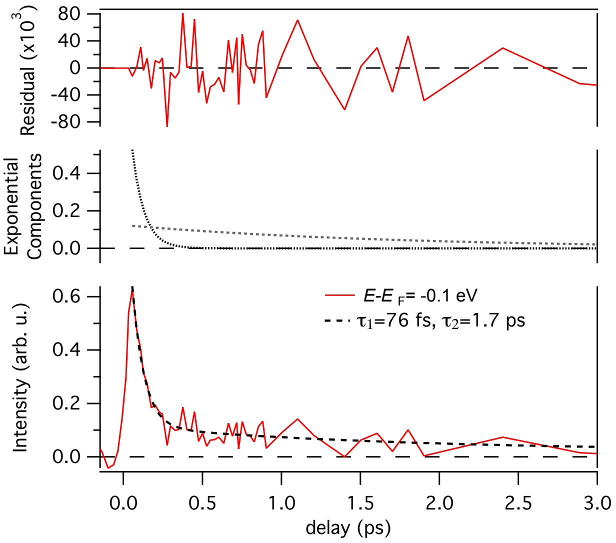

Supplementary Figure 4: Fit procedure for the time-resolved PhotoElectron spectroscopy time evolution The fitting procedure requires a double exponential function. The two component are represented as well as the residual (difference between the data and the fitting function).

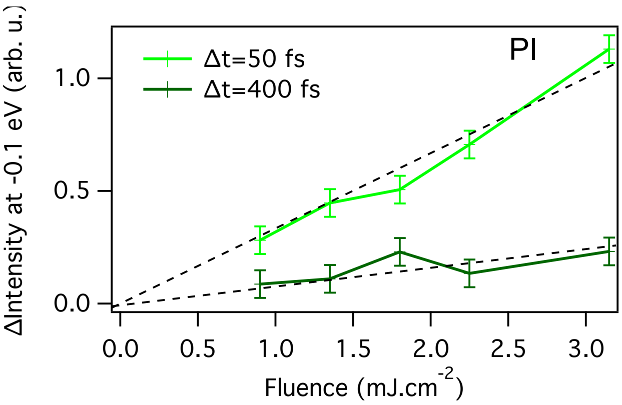

Supplementary Figure 5: Time-resolved PhotoElectron spectroscopy Intensity at 50 fs and 400 fs as a funciton of fluence. The intensity differences were taken at -0.1 eV and they show a linear behavior with fluence. The error bars are the standard deviation calculated using the number of shots on the detector.

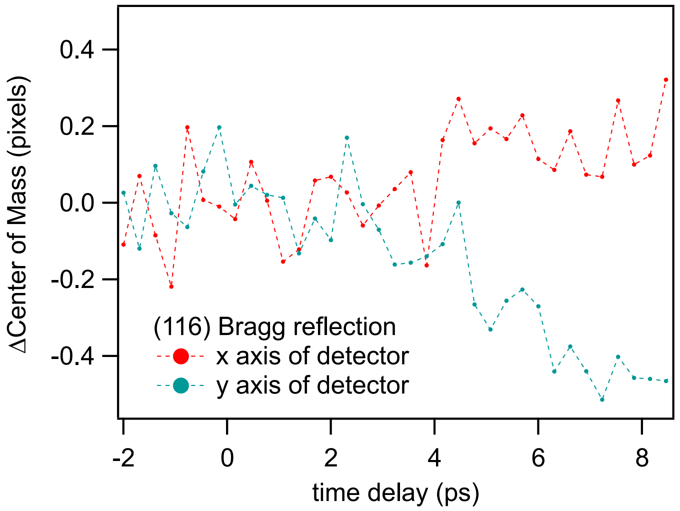

Supplementary Figure 6: Time-resolved X-ray diffraction on the (116) Bragg reflection. The center of mass of the peak signal on the detector shows a shift after 4 ps, corresponding to the onset of the acoustic wave propagation.

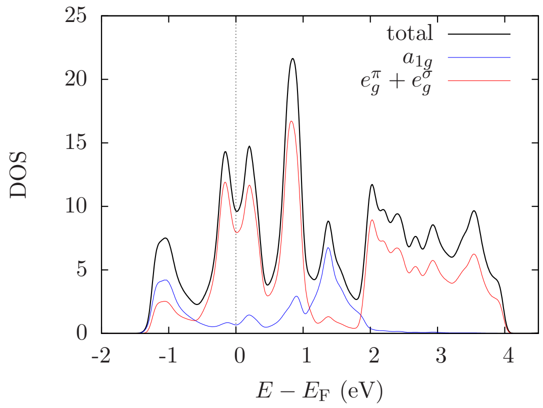

Supplementary Figure 7: GGA Calculations. GGA-density of states of V_2O_3 projected onto atomic vanadium d-orbitals.

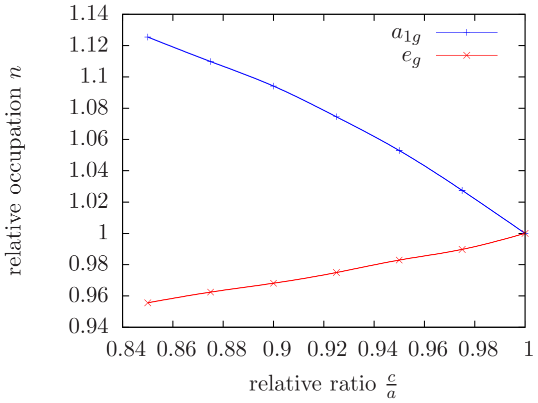

Supplementary Figure 8: Orbital occupation of each orbital as a function of the ratio c/a. Each occupancy is measured relatively to its value at equilibrium, i.e. at $c/a = 1$.

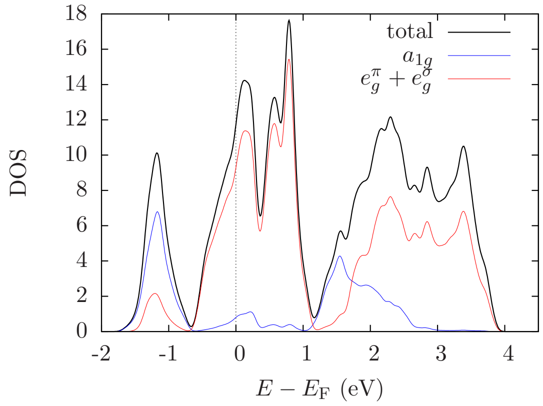

Supplementary Figure 9: GGA Calculations with *ca=0.85*

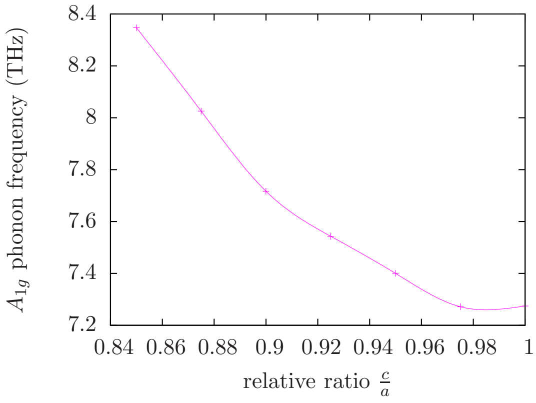

Supplementary Figure 10: Obtained A_{1g} phonon frequencies as a function of the ratio *c/a*

Supplementary Note 1. Temperature dependence in Photoelectron spectroscopy

In order to distinguish a non-thermal phase from a thermal one, photoemission spectra versus temperature were taken using the same experimental setup as for trPES, but without a pump pulse. Supplementary Figure 1 shows the temperature evolution of the paramagnetic metallic phase (PM) versus temperature. The quasiparticle weight (Intensity between -0.4 and 0 eV) shows a linear behavior in the range between 200 and 300 K, consistently with previous results [2], whereas the spectral weight above the Fermi level seems constant within our energy resolution, which is 70 meV. In the PI phase the temperature difference is also linear in the considered temperature range, see figure 2. These spectral differences vs temperature are therefore good references for the out of equilibrium ones.

Supplementary Note 2. Synchrotron radiation based X-ray diffraction experiments

Before the experiments with sub-ps time resolution at the LCLS, we performed an extensive series of trXRD measurements at synchrotron radiation facilities, using the storage ring temporal structure with a time resolution of 100 ps. This allowed us to precisely evaluate thermal effects for the system's behavior at long time delays. The experiments were performed on beamline 6.0.1 at the ALS (Berkeley, USA) and on the Cristal Beamline at SOLEIL (Saint-Aubin, France). We used a 1° incidence angle for the X-rays and a normal incidence angle for the 1.5 eV optical pump, at a fluence of 5 mJ.cm^{-2}. The X-ray energy was 6 keV, which enables perfect penetration depth overlap with the optical pump. The measurements were done at 1 kHz repetition rate. The Bragg reflections were monitored using a point detector.

The evolution of two quantities was monitored, namely the center of mass of the rocking curve and its intensity (Supplementary Figure 3). The combination of the 3 Bragg reflection intensities can unambiguously disentangle the

2 Wyckoff positions and the Debye-Waller factor[3]. The center of mass can also be used to calculate the new lattice parameters supposing that only the value of the a and c lattice parameters change. We find that the Wyckoff positions change gives a temperature rise of 25 K using the tabulated values from ref [4], while the change of the rocking curve position gives a temperature rise of 19 K (using the lattice expansion coefficients from ref [5]) (the latter approach is less precise, but the two values agree). The probing depths of trPES and of trXRD are considerably different (3 nm for trPES and 90 nm for trXRD): Therefore for a same fluence the average density excitation is different, since the excitation has an exponential attenuation along the depth of the sample. The estimated difference for the effective excitation in the two cases is a factor 2, therefore a fluence of 5 mJ.cm^{-2} in trXRD is equivalent to 2.5 mJ.cm^{-2} in trPES. This allows us to conclude that the temperature increase in the trPES experiments should be around 20 K. The trPES behavior is also linear with fluence, see Supplementary Figure 5, which indicates that results from TRR and trXRD can be safely compared with the trPES experiment.

Supplementary Note 3. Fitting procedure

We use the built in fitting procedure from Igor pro software which uses a least square method. The double exponential fit was used in order to capture two timescales from the trPES time traces. Supplementary Figure 4 shows an example of fitting.

Supplementary Note 4. FEL based experiments at the LCLS

For the trXRD experiment at LCLS, both the center of mass of the Bragg reflection on the detector and its intensity were monitored. The evolution of the latter is presented in the main text and is related directly to the structural dynamics before 4 ps since the center of mass does not move. The former does not change before 4 ps as shown in Supplementary Figure 6: this proves that the unit cell does not change during the first 4 ps, while afterwards the thermal expansion becomes the dominant effect.

Supplementary Note 5. Theory of light-induced A_{1g} phonon hardening

In order to support the idea of phonon hardening due to an increase of the occupation of the a_{1g} orbitals in the trigonal crystal field splitting, density functional theory (DFT) calculations within the generalized gradient approximation, [6] (GGA) using the Quantum ESPRESSO [7] code. The ultrasoft pseudopotential formulation, pseudopotentials `V.pbe-n-van.UPF` and `O.pbe-van_ak.UPF` from the Quantum ESPRESSO pseudopotential data base [8], has proven to be useful in the context of 3d elements like V. Concerning technical details, a Monkhorst-Pack 21x21x21 $\underline{k}$-point mesh (891 points in the irreducible wedge) and a plane-wave cutoff of 30 Ry for wave functions and 240 Ry for charge densities has been found to be sufficient.

In its paramagnetic phases, V_2O_3 crystallizes in the corundum structure, requiring four V atoms per primitive unit cell. Structural data (hereafter referred to as "experimental") are taken from Ref. 9. The Wyckoff positions of the atoms in the unit cell have been found by relaxation within GGA, which is a necessary step for the subsequent phonon calculation.

Supplementary Figure 7 shows the obtained Kohn-Sham density of states for the experimental unit cell. It agrees well with previously published data. Note that due to the relaxation within GGA, small deviations from experimental data occur, especially the less pronounced gap between t_{2g} and e_g^σ. The projections onto atomic vanadium d-orbitals clarify the distribution of a_{1g} and e_g spectral weight and show nicely the bonding-antibonding splitting of the $a_{1g} \sim d_{z^2}$ orbitals, which strongly hybridize along the z-axis. As it is well known, GGA cannot describe the insulating phase, even allowing for magnetism and monoclinic distortion towards which the undistorted paramagnetic solution is unstable [10]. An insulating phase with the magnetic structure and lattice distortion actually observed in V_2O_2 at low temperatures can be stabilised only within GGA+U [10], through the emptying of the a_{1g} orbital driven by the correlation enhanced crystal field splitting as originally proposed by Ref. [11]. However, in order not to face any issue related to double-counting in GGA+U, we decided to stay within straight GGA, hence limit our analysis to the paramagnetic undistorted metal phase.

Assuming that the main effect of the pump laser is to increase the a_{1g} occupancy, we need to model it by an auxiliary effect that suitably modifies the occupation, while influencing the overall electronic structure as few as possible. It has been shown [12] that the modification of the ratio c/a of the hexagonal structural parameters, while fixing the unit cell

volume at their experimental value and relaxing the Wyckoff positions within GGA, might be suitable. The obtained relationship thereof is shown in Supplementary Figure 8. The occupation values are taken from the LDA+U basis of QUANTUM ESPRESSO (without actually adding U), which comprises atomic-like vanadium d-orbitals, identifying a_{1g} with d_{z^2}. It shall be mentioned that due to their specific localization, this basis does not exclusively capture spectral d-weight, but also e.g. contributions from neighboring atoms. However, the desired behavior is indeed recovered: the a_{1g} occupation increases at expenses of the e_g one as c/a diminishes.

In order to support the statement that the electronic structure is otherwise not heavily affected by the "squeezing" of the unit cell, Supplementary Figure 9 shows the projected density of states at the maximal case of $c/a = 0.85$. One can still identify the bonding-antibonding feature of the a_{1g}-orbitals, though the bonding part now carries significantly more spectral weight. Major changes can be seen merely in the unoccupied spectral range, where the gap between t_{2g} and e_g^σ diminishes.

Using QUANTUM ESPRESSO, it is straightforward to calculate phonon frequencies in the framework of density functional perturbation theory (see e.g. Ref. [13]). Among the phonons with A_{1g} symmetry at the Γ-point that can be obtained within this procedure, one (i.e. the lowest) can be identified as the phonon in question. Its frequency as a function of relative c/a ratio is drawn in Supplementary Figure 10. It shows the qualitative behavior that we anticipated, however in the experiment the unit cell is fixed and only the Wyckoff positions change. Therefore, although our calculation is done at equilibrium, still it suggests that an increase of the a_{1g} population may drive a blue-shift of the A_{1g} mode, as indeed observed in the experiment.

Supplementary references

[1] Misochko, O. *et al.* Optical study of the Mott transition in V_2O_3: Comparison of time- and frequency-domain results. *Physical Review B* **58**, 12789–12794 (1998).

[2] Rodolakis, F. *et al.* Quasiparticles at the Mott Transition in V_2O_3: Wave Vector Dependence and Surface Attenuation. *Physical Review Letters* **102**, 066805 (2009).

[3] Johnson, S., Vorobeva, E., Beaud, P., Milne, C. & Ingold, G. Full Reconstruction of a Crystal Unit Cell Structure during Coherent Femtosecond Motion. *Physical Review Letters* **103**, 205501 (2009).

[4] Robinson, W. R. High-temperature crystal chemistry of V_2O_3 and 1% Chromium-doped V_2O_3. *Acta Crystallographica Section B* **31**, 1153 (1975).

[5] McWhan, D. B. & Remeika, J. P. Metal-Insulator Transition in $(V_{1-x}Cr_x)_2O_3$. *Physical Review B* **2**, 3734 (1970).

[6] Perdew, J. P., Burke, K. & Ernzerhof, M. Generalized gradient approximation made simple. *Phys. Rev. Lett.* **77**, 3865–3868 (1996).

[7] Giannozzi, P. *et al.* Quantum espresso: a modular and open-source software project for quantum simulations of materials. *Journal of Physics: Condensed Matter* **21**, 395502 (2009).

[8] Quantum espresso: Pseudopotentials. http://www.quantum-espresso.org/pseudopotentials. (2009).

[9] Dernier, P. D. & Marezio, M. The crystal structure of V_2O_3 and $(V_0.962Cr_0.0382)_2O_3$ near the metal-insulator transition. *Journal of Physics and Chemistry of Solids* **2**, 3771–3776 (1970).

[10] Grieger, D. & Fabrizio, M. Low-temperature magnetic ordering and structural distortions in vanadium sesquioxide V_2O_3. *Phys. Rev. B* **92**, 075121 (2015).

[11] Poteryaev, A. *et al.* Enhanced crystal-field splitting and orbital-selective coherence induced by strong correlations in V_2O_3. *Physical Review B* **76**, 085127 (2007).

[12] Grieger, D. & Lechermann, F. Effect of chromium doping on the correlated electronic structure of V_2O_3. *Phys. Rev. B* **90**, 115115 (2014).

[13] Baroni, S., de Gironcoli, S., Dal Corso, A. & Giannozzi, P. Phonons and related crystal properties from density-functional perturbation theory. *Rev. Mod. Phys.* **73**, 515–562 (2001).
